# Successful Treatment of Granuloma Faciale With Deep Dermal Inflammatory Infiltration Using Intralesional Corticosteroid Injections: A Case Report

**DOI:** 10.7759/cureus.111010

**Published:** 2026-06-17

**Authors:** Mioka Homma, Tatsuya Katsumi, Ryoya Ohashi, Shota Uchida, Riichiro Abe

**Affiliations:** 1 Department of Dermatology, Faculty of Medicine, Graduate School of Medical and Dental Sciences, Niigata University, Niigata, JPN

**Keywords:** corticosteroid injection, granuloma faciale, pathological features, successful treatment, treatment choices

## Abstract

A 46-year-old man presented with asymptomatic dark reddish nodules on the left nasal ala and lower jaw, including a 1-cm nodule on the nasal ala. Histopathological examination revealed diverse inflammatory cell infiltration extending into the deep dermis and the presence of a grenz zone. Based on these findings, he was diagnosed with granuloma faciale (GF). Intralesional corticosteroid injections were selected because of their strong anti-inflammatory effects and efficacy in treating deep dermal lesions. Although various treatment modalities have been attempted, GF is often refractory to treatment. In this case, the lesion resolved completely after three intralesional corticosteroid injections.

## Introduction

Granuloma faciale (GF) is a rare, benign inflammatory dermatosis that typically presents as a solitary, well-demarcated reddish-brown to violaceous papule, nodule, or plaque with follicular accentuation and telangiectasia, most commonly involving the face, particularly the forehead, cheeks, and nose [1,2]. It has a slight male predominance [2]. Diagnosis is established by skin biopsy, which is essential for excluding other dermatologic conditions with similar clinical features, including rosacea, sarcoidosis, lupus vulgaris, fungal infections, mycobacterial infections, and discoid lupus erythematosus [3]. GF is characterized histopathologically by a grenz zone and diverse inflammatory cell infiltration [4]. Various treatment modalities have been reported, including topical corticosteroids, topical tacrolimus, intralesional corticosteroids, and laser therapy, although treatment responses are often variable [2]. Here, we report a case of localized GF with deep dermal inflammatory infiltration involving the nasal ala and lower jaw that responded completely to intralesional triamcinolone acetonide injections.

## Case presentation

**Figure 1:**
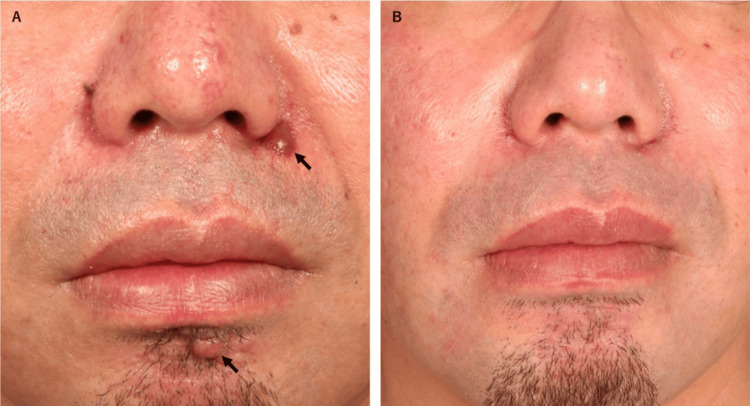
Clinical features (A) An asymptomatic dark red nodule on the left nasal ala and lower jaw (arrows indicate the lesions). (B) Posttreatment appearance showing complete resolution after intralesional corticosteroid injection.

A 46-year-old man with no significant past medical or family history developed a reddish-brown nodule on the lateral surface of the left nasal ala seven weeks before his first visit to our department. Treatment with oral doxycycline and topical nadifloxacin for two weeks showed no improvement, and subsequent treatment with oral minocycline for four weeks also failed to improve the condition. On examination, we found an asymptomatic dark red nodule, 1 cm in diameter, on the lateral aspect of the left nasal ala. A similar nodule was also present on the lower jaw (Figure 1A).

**Figure 2:**
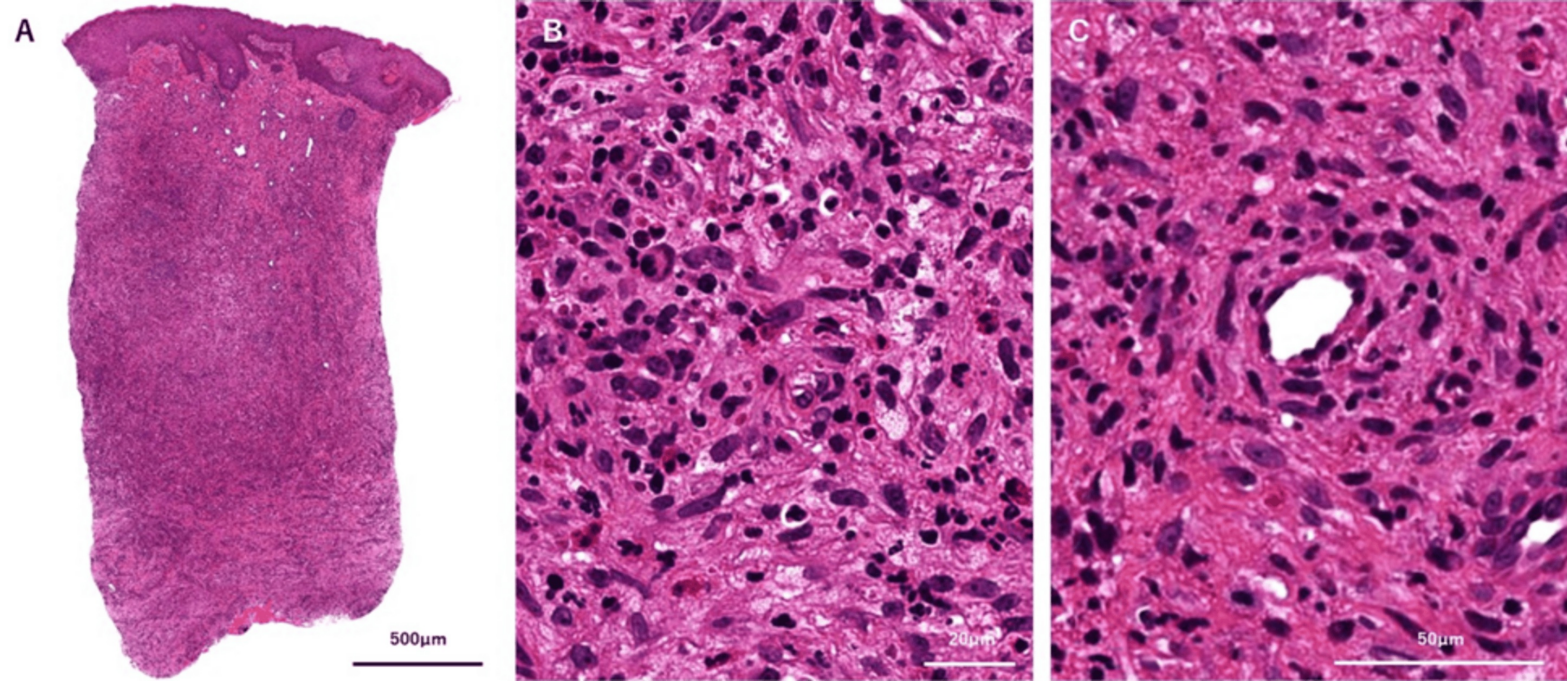
Histopathological findings of skin biopsy from the left nasal ala (hematoxylin and eosin stain) (A) Histopathology at low magnification showing a subepidermal grenz zone and dense dermal inflammatory infiltrates. (B) Histopathology at high magnification showing mixed inflammatory infiltrates. (C) No evidence of vasculitis was observed.

A skin biopsy was performed on the nasal ala lesion. Histopathological analysis revealed a mild subepidermal grenz zone and a dense polymorphous inflammatory infiltrate extending throughout the dermis into the deep dermis (Figure 2A). The infiltrate was composed of lymphocytes, histiocytes, plasma cells, neutrophils, and scattered eosinophils, although eosinophils were not a prominent component (Figure 2B). No granulomatous changes were observed. Mild leukocytoclasia was present but not prominent. Furthermore, vessel wall damage and fibrin deposition were absent, and no definite vasculitic changes were identified (Figure 2C). No atypical lymphoid infiltrates were identified. Ziehl-Neelsen staining was negative for acid-fast bacilli. These findings were consistent with GF. A biopsy was also performed on the lower jaw lesion, and histopathological examination revealed findings similar to those observed in the nasal ala lesion. Based on the presence of a grenz zone and diverse inflammatory infiltrates, a diagnosis of GF was made.

Histopathological examination revealed extensive inflammatory cell infiltration extending into the deep dermis. Intralesional corticosteroid injections were selected because of their potent anti-inflammatory properties and established use in dermatologic conditions requiring deep tissue penetration. A mixture of 1 mL of triamcinolone acetonide suspension (40 mg/mL) and 1 mL of 1% lidocaine with epinephrine (1:100,000) was prepared, resulting in a final triamcinolone concentration of 20 mg/mL. Three injections were administered at four-week intervals. A total volume of 2 mL was injected per session, with approximately 1 mL injected directly into each nodule (total triamcinolone dose, 40 mg per session). Both lesions were treated at each session. The most marked clinical improvement was observed after the first injection, followed by gradual further regression after the subsequent treatments. The lesions gradually regressed following the initiation of treatment, and complete resolution was achieved approximately six months after the first injection (Figure 1B). No adverse effects, such as skin atrophy or hypopigmentation, were observed during the treatment course. The patient was followed for three months after complete resolution, during which no recurrence was observed.

## Discussion

Regarding the diagnosis, GF was suspected based on the presence of diverse cellular infiltration and a grenz zone [5]. Vascular changes were frequently observed [5]. However, many reports have indicated that clear vasculitis is not always observed in GF [6]. Previous reports have described vasculitis in only two of 11 cases and in five of 73 cases, suggesting that its presence is not essential for diagnosis [5,7]. The most frequent histopathologic features of GF were the presence of a grenz zone, neutrophilic infiltration, and telangiectasia [5]. GF is therefore often diagnosed based on diverse cellular infiltration and a grenz zone alone, without mandatory evidence of vasculitis [5]. In our case, a diagnosis of GF was made based on the presence of a grenz zone and a diffuse inflammatory cell infiltrate in the dermis, including neutrophils. The absence of granulomatous inflammation, atypical lymphoid infiltrates, and acid-fast bacilli helped exclude sarcoidosis, infectious granulomatous diseases, and lymphoma/pseudolymphoma, whereas the characteristic grenz zone and polymorphous inflammatory infiltrate further supported the diagnosis of GF.

Various treatments have been reported for GF, including topical tacrolimus, systemic therapies, and several forms of laser treatment, each with varying degrees of success [2]. For example, topical tacrolimus and laser treatments have been reported to show relatively high success rates [2]. However, because of the chronic and treatment-resistant nature of the disease, the optimal approach remains unclear [2]. Intralesional corticosteroids are often favored because they allow direct delivery of the drug into the deeper dermis, where inflammatory infiltrates typically reside. In this case, given the depth of the inflammatory infiltrate, topical agents were considered less likely to be effective because their penetration is generally limited to the superficial dermis. Therefore, intralesional corticosteroid injections were selected to deliver therapeutic effects directly to the deeper dermis.

## Conclusions

We report a case of GF successfully treated with intralesional triamcinolone acetonide injections. This therapeutic option may be particularly effective when lesions extend into the deep dermis. Our case supports previous reports demonstrating the usefulness of intralesional triamcinolone acetonide as a practical treatment for GF. Further accumulation of cases will be needed to clarify the role of intralesional corticosteroids among the available treatment options for this challenging condition.

## References

[1] Wigley JE (1945). Eosinophilic granuloma.? Sarcoid of Boeck. Proc R Soc Med.

[2] Lindhaus C, Elsner P (2018). Granuloma faciale treatment: a systematic review. Acta Derm Venereol.

[3] Frankel DH, Soltani K, Medenica MM, Rippon JW (1988). Tinea of the face caused by Trichophyton rubrum with histologic changes of granuloma faciale. J Am Acad Dermatol.

[4] Kaur M, Singh A, Ramesh V (2016). Granuloma faciale. Indian Dermatol Online J.

[5] Ortonne N, Wechsler J, Bagot M, Grosshans E, Cribier B (2005). Granuloma faciale: a clinicopathologic study of 66 patients. J Am Acad Dermatol.

[6] Zirwas MJ, Abell E, Ruben A, Silverman AR, Wolff J, Deng JS (2003). Immunofluorescence findings in granuloma faciale: report of two cases. J Cutan Pathol.

[7] Marcoval J, Moreno A, Peyr J (2004). Granuloma faciale: a clinicopathological study of 11 cases. J Am Acad Dermatol.

